# Effects of *Boswellia serrata* on cell viability, scratch wound closure, and gene expression in human gingival fibroblasts

**DOI:** 10.1016/j.jobcr.2026.101537

**Published:** 2026-09-10

**Authors:** Shima Golmohammadi, Zahra Daneshi, Media Anousheh

**Affiliations:** Department of Periodontics, Faculty of Dentistry, Islamic Azad University, Borujerd Branch, Modarres Square, Borujerd, Lorestan Province, Iran

**Keywords:** *Boswellia*, Plant extracts, Fibroblasts, Wound healing, Gene expression, Extracellular matrix, Cell survival, Cell movement

## Abstract

**Objectives:**

*Boswellia serrata*, traditionally used for treating inflammatory conditions, has demonstrated anti-inflammatory and antimicrobial properties, but its direct effects on human gingival fibroblasts (HGFs) remain unexplored. Therefore, the aim of this study was to evaluate the effects of methanolic *B. serrata* extract on human gingival fibroblast cell viability and scratch wound closure and to analyze the expression of key wound healing-related genes.

**Materials and methods:**

HGF-1 cells were treated with various concentrations of *B. serrata* extract. Cell viability was assessed using MTT assay, while in vitro scratch assay was used to evaluate wound closure. Additionally, expression of COL1A1, fibronectin (FN1), TGF-β, and VEGF genes were analyzed by quantitative reverse transcription polymerase chain reaction (qRT-PCR).

**Results:**

Concentrations up to 10 μg/mL maintained cell viability above 80% for 72 h. The scratch assay demonstrated significant enhancement of wound closure at 2.5, 5, and 10 μg/mL compared to control (p = 0.012), with the highest numerical wound-closure value observed at 2.5 μg/mL (99.9%). qRT-PCR revealed significant upregulation of COL1A1 (2.68-fold), FN1 (2.12-fold), and VEGF (3.54-fold) (P < 0.001), while the increase in TGF-β expression was non-significant (1.74-fold, P = 0.491).

**Conclusion:**

*Boswellia serrata* extract demonstrated favorable effects on HGF-1 cell viability, scratch wound closure, and wound healing-related gene expression at non-cytotoxic concentrations. These findings support further preclinical investigation of *B. serrata* as a potential adjunctive agent for periodontal wound healing and regeneration.

## Graphical abstract

**Figure:**
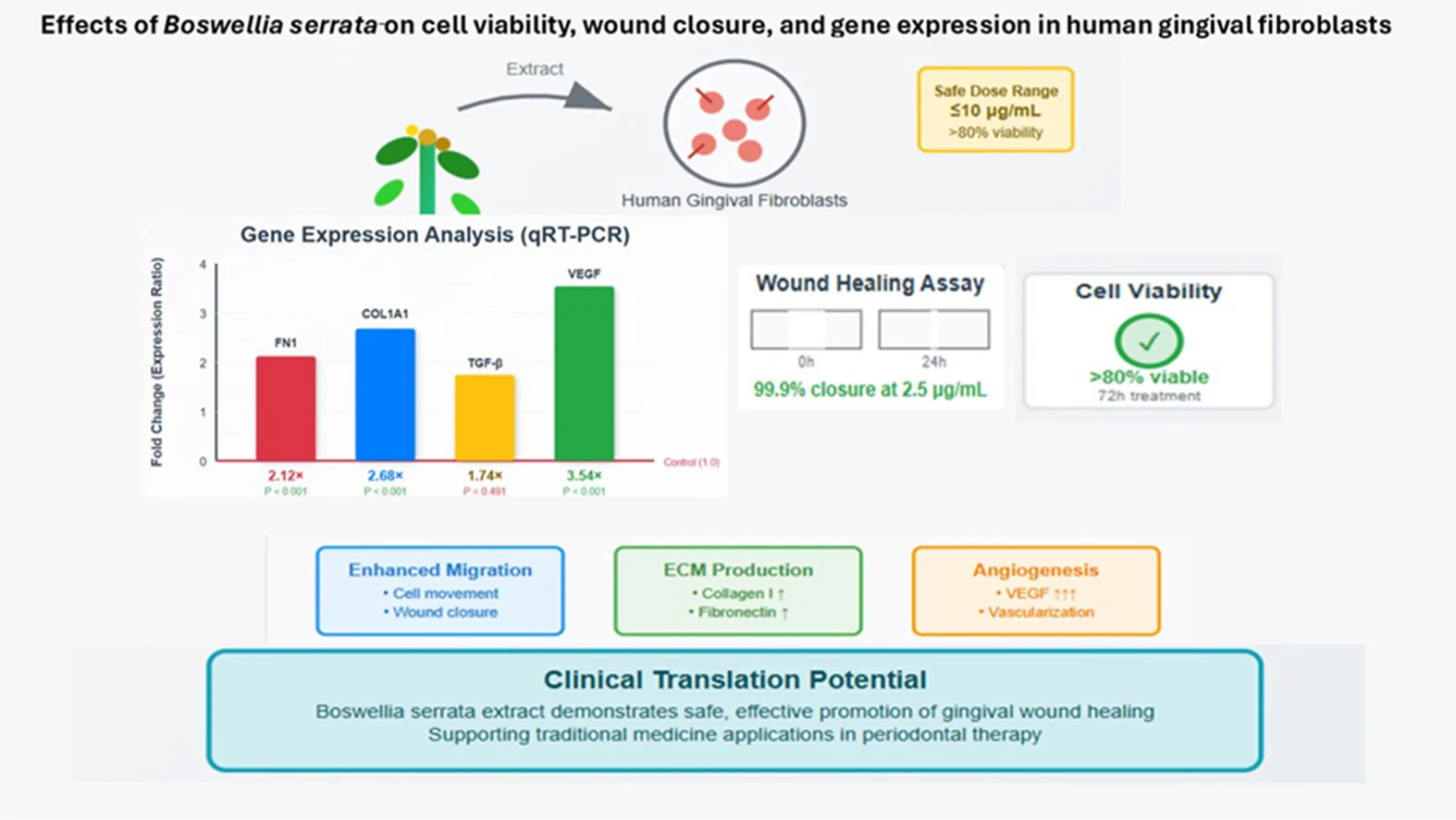

## 1 Introduction

Wound healing in the oral cavity and gingival tissues is a complex biological process requiring coordinated cellular proliferation, migration, and extracellular matrix (ECM) synthesis. After the initial infiltration of platelets and inflammatory cells, fibroblasts are among the first cells to repopulate the gingival wound site.^1^ These cells are the predominant type of gingival connective tissue cells and play a central role in healing by migrating into damaged areas, proliferating, and producing key ECM components essential for tissue repair.^2^ Wound closure requires fibroblast migration along wound edges and their proliferation to restore tissue integrity. Fibrillar collagen (types I/III), fibronectin, and other ECM components produced by fibroblasts in the gingival lamina propria form a structural scaffold needed for cellular migration and tissue organization.^3^

Additionally, HGFs secrete critical signaling molecules such as transforming growth factor-beta (TGF-β), vascular endothelial growth factor (VEGF), and platelet-derived growth factor (PDGF) that promote angiogenesis, immune cell recruitment, and epithelial proliferation.^2^

At the molecular level, wound healing regulation involves coordinated expression of key genes including type I collagen (COL1A1), which provides structural integrity to the healing tissue matrix^4^; fibronectin (FN1), which facilitates cellular adhesion and migration^5^; VEGF, mediator of angiogenesis^6^; and TGF-β, regulator of fibroblast activation and ECM production.^7^ These genes serve as reliable indicators of tissue regenerative capacity and therapeutic efficacy.^8^

*Boswellia serrata*, also known as Indian frankincense, has long been used in traditional medicine to treat inflammatory conditions and promote wound healing. Its therapeutic properties are primarily attributed to boswellic acids, which reduce inflammation through inhibition of 5-lipoxygenase (thereby reducing leukotriene synthesis, a key mediator in chronic inflammatory processes including periodontitis), inhibition of leukocyte elastase (enzyme that initiates tissue damage and triggers inflammatory cascade), and suppression of nuclear factor-κB (NF-κB) pathways, while also demonstrating significant antioxidant properties.^9^, ^10^, ^11^.

Recent studies have highlighted *B. serrata*'s potential in oral health applications. The extract has demonstrated antibacterial and antibiofilm activities against periodontal pathogens such as *Porphyromonas gingivalis*.^12^ Traditional applications in various cultures include strengthening teeth and gums, treating inflammatory oral conditions, and promoting wound healing, suggesting its potential therapeutic value in periodontal therapy.^9^^,^^13^

In periodontal disease, chronic inflammation leads to tissue damage and impaired wound healing. Leukotrienes contribute to this process through activation of inflammatory cells and tissue destruction.^14^ The ability of *B. serrata* to modulate leukotriene pathways and regulate immune responses makes it relevant for periodontal applications, particularly in enhancing fibroblast-mediated tissue repair.^15^

Despite extensive documentation of *B. serrata*'s traditional therapeutic applications and well-established anti-inflammatory properties,^9^ there remains a significant knowledge gap regarding its direct effects on human gingival fibroblasts (HGFs) at the cellular and molecular levels. While research has primarily focused on systemic inflammatory conditions, the effects on HGFs—critical cells in periodontal wound healing—remain unexplored. Given that periodontitis, gingivitis, and most oral wounds are fundamentally inflammatory in nature, understanding *B. serrata*'s specific effects on HGFs becomes particularly relevant for oral therapeutic applications.

The pharmacological activities of boswellic acids have been demonstrated to modulate diverse targets such as enzymes, growth factors, kinases, and transcription factors,^16^ yet no studies have specifically investigated how these mechanisms translate to HGF behavior and periodontal tissue regeneration. Recent oral health research with *B. serrata* has been limited to antimicrobial effects on periodontal pathogens such as *Porphyromonas gingivalis* in gingival epithelial cells,^17^ leaving the fundamental question of its impact on fibroblast viability, scratch wound closure, and wound healing-related gene expression unexplored. Given that human gingival fibroblasts are undifferentiated cells with multi-differentiation and self-renewal capacities that play crucial roles in wound healing,^18^ understanding *B. serrata*'s specific effects on these cells and their molecular responses is essential for developing evidence-based therapeutic protocols for periodontal regeneration and oral wound healing. Therefore, this study aimed to evaluate the effects of methanolic *B. serrata* extract on HGF-1 cell viability, assess its effects on scratch wound closure using an in vitro scratch model, and analyze the expression of key wound healing-related genes (FN1, COL1A1, TGF-β, and VEGF) using quantitative reverse transcription PCR.

## 2 Materials and Methods

This in vitro experimental study was approved by the appropriate institutional ethics committee (IR.IAU.B.REC.1402.078).

## 3 Preparation of methanolic extract of *Boswellia serrata*

The oleo-gum-resin of *B. serrata* was purchased from a certified herbal medicine supplier with established quality control procedures. A voucher specimen number was not available for the material used in this study. The gum was ground into a fine powder, and the extract was prepared using the soaking method. Approximately 100 g of powdered resin was placed in an Erlenmeyer flask with 1 L of 80% methanol (v/v) at a 1:10 (w/v) ratio. The mixture was stirred thoroughly and kept at room temperature for 24 h in darkness. After incubation, the solution was filtered through Whatman filter paper. The filtrate was concentrated under reduced pressure at 40°C using a rotary evaporator (BÜCHI® GmbH) at 40 rpm and 65 mbar to remove methanol without degrading heat-sensitive compounds. The remaining plant material underwent repeated extractions with fresh 80% methanol for 48, 72, 96, and 120 h, to maximize yield. After each incubation, filtration and concentration were performed as described above. The combined extracts were pooled and dried completely under a fume hood. The resulting crude extract was stored at 4°C in an airtight container until analysis. The resulting methanolic extract was evaluated as a crude extract and was not subjected to chemical standardization or quantitative characterization of individual constituents, including boswellic acids.

## 4 Cell culture and MTT cytotoxicity assay

Human gingival fibroblasts (HGF-1) were obtained from a national cell bank (NCBI code: C165; Pasteur Institute) and cultured in Roswell Park Memorial Institute (RPMI-1640) medium (Gibco, Thermo Fisher Scientific) supplemented with 10% fetal bovine serum (FBS) (Gibco, Thermo Fisher Scientific). For the assay, cells (10,000 cells/well) were seeded into 96-well plates (Nunc™, Thermo Fisher Scientific). After 18 h, various concentrations of *B. serrata* extract were added to the wells and cell viability was evaluated using the MTT assay at 24, 48, 72 and 96 h. The MTT assay was performed in three independent experiments using independently prepared HGF-1 cultures. Within each independent experiment, each concentration was assessed in three technical replicate wells. MTT stock solution (5 mg/mL; Sigma-Aldrich) was added at 10% of culture volume, and plates were incubated for 4 h at 37°C in a CO_2_ incubator (Biotek Instruments). The supernatant was aspirated, and formazan crystals were dissolved in 100 μL of dimethyl sulfoxide (DMSO; Merck). Absorbance was measured at 570 nm to quantify the formazan product and at 630 nm to correct for background absorbance using a microplate spectrophotometer (Epoch, BioTek Instruments). Net absorbance was calculated by subtracting the 630 nm reading from the 570 nm reading. Cell viability was expressed as percentage relative to untreated controls (100%). Cell growth inhibition was calculated using the formula:

Growthinhibition%=(1−TestV/MaxV)×100

where *Test V* is the absorbance of treated cells and *Max V* is the control absorbance. The half-maximal inhibitory concentration (IC50) was determined by fitting dose-response curves using non-linear regression analysis in GraphPad Prism software (version 8.0).

## 5 In vitro wound healing (Scratch) assay

To evaluate the effect of *B. serrata* on in vitro wound closure in human gingival fibroblasts, a scratch assay was performed. HGF-1 cells were seeded into 4-well plates at a density of 100,000 cells per well (26,316 cells/cm^2^). Culture medium was Dulbecco's Modified Eagle Medium (DMEM) supplemented by 10% FBS, 1% penicillin-streptomycin, 2 mM L-glutamine, 1 mM sodium pyruvate. Wells with 10% and 1% FBS served as positive and negative controls, respectively. After 24 h, when the cells reached near-confluence, a sterile 1 mL pipette tip created a straight scratch line across the monolayer. Debris was removed by washing with phosphate-buffered saline (PBS). Cells were treated with *B. serrata* extract at 0, 2.5, 5, and 10 μg/mL immediately after scratching (time 0) and incubated at 37°C with 5% CO_2_. Images were captured at 0 and 24 h using an inverted microscope (Eclipse TS100, Nikon) with digital camera. Wound areas were quantified using ImageJ software (version 1.45S, Wayne Rasband, National Institutes of Health). The scratch assay was performed in three independent experiments using independently prepared HGF-1 cultures, with three technical replicate wells per experimental condition in each independent experiment. Percentage of wound closure was calculated as: [(Initial wound area at 0 h – Wound area at 24 h)/Wound area at 0 h] × 100.

## 6 Quantitative reverse transcription polymerase chain reaction (qRT-PCR)

Quantitative RT-PCR was used to assess the expression of genes associated with extracellular matrix production: type I collagen (COL1A1), fibronectin (FN1), transforming growth factor-beta (TGF-β), and vascular endothelial growth factor (VEGF). HGF-1 cells were seeded at 10,000 cells/well and treated with *B. serrata* extract at 2.5, 5, and 10 μg/mL.

Total RNA was extracted using TRIzol™ Reagent (Cat. No. 2302700; Prime). Chloroform (100 μL) was added for phase separation, followed by centrifugation at 12,000 rpm for 15 min. RNA was precipitated with 250 μL isopropanol, washed and dissolved in nuclease-free water. RNA concentration and purity were assessed using a NanoDrop™ 2000 spectrophotometer (Thermo Scientific). First-strand complementary DNA (cDNA) synthesis was performed using the ImProm-II™ Reverse Transcription System (Cat. No. A3800; Promega). qPCR was conducted using gene-specific primers. Gene expression levels were normalized to B2M (beta-2 microglobulin) as the housekeeping gene, which served as an internal control due to its stable expression. Relative quantification was calculated using the comparative Ct method (ΔΔCt). The Ct (cycle threshold) value represents the PCR cycle number at which the fluorescence signal exceeds a defined threshold, reflecting the amount of target gene expression. This method compares Ct values of target genes normalized to the housekeeping gene and relative to the control sample. Fluorescence data were processed using Bio-1D software (version 15.03; Vilber Lourmat). The qRT-PCR analysis was performed in three independent experiments using independently prepared HGF-1 cultures, with each qPCR reaction performed in technical triplicate. Technical replicate Ct values were averaged within each independent experiment. Gene expression was normalized to B2M, and relative expression was calculated using the comparative Ct (ΔΔCt) method and expressed as fold change relative to the untreated control.

## 7 Statistical analysis

Data are presented as mean ± standard deviation (SD) from three independent experiments (n = 3). Within each experiment, technical replicates were averaged and were not treated as independent observations. Data normality was assessed using the Shapiro–Wilk test before applying parametric analyses. For the MTT assay, data were analyzed using two-way ANOVA, with extract concentration and incubation time as factors, including assessment of the concentration × time interaction, followed by Tukey's multiple-comparisons test. Because different wells were evaluated at each time point, the data were not treated as repeated measures. For the scratch assay, differences among treatment groups were analyzed using one-way ANOVA followed by Tukey's post hoc test. A value of p < 0.05 was considered statistically significant. Statistical analyses for the MTT and scratch assays were performed using GraphPad Prism software (version 8.0). For qRT-PCR, statistical significance was assessed using normalized Ct-derived gene-expression data by comparing treated samples with the untreated control. The corresponding software-generated P(H1) values are reported in Table 1. Fold-change values were used to present relative expression and were not directly used for statistical testing.

**Table 1:** **Relative expression of wound healing-related target genes in human gingival fibroblasts treated with *B. serrata* extract.** Expression levels were normalized to the reference gene B2M and calculated using the ΔΔCt method. Relative expression is presented as fold change with standard error and 95% confidence interval. P(H1) represents the software-generated significance value for the comparison of normalized target-gene expression between treated samples and the untreated control. P(H1) values < 0.05 were considered statistically significant. “UP” indicates statistically significant upregulation compared with the untreated control.

Gene	Type	Reaction Efficiency	Expression	Std. Error	95% C.I.	P(H1)	Result
B2M	REF	1.0	1.000				
FN1	TRG	1.0	2.121	1.120 - 4.032	1.067 - 4.222	0.000	UP
COL1A1	TRG	1.0	2.676	2.468 - 2.905	2.393 - 2.994	0.000	UP
TGF-β	TRG	1.0	1.741	1.093 - 2.850	0.947 - 3.224	0.491	
VEGF	TRG	1.0	3.543	1.792 - 7.741	1.341 - 9.633	0.000	UP

## 8 Results

### 8.1 Cell viability and IC50 determination

**Fig. 1:**
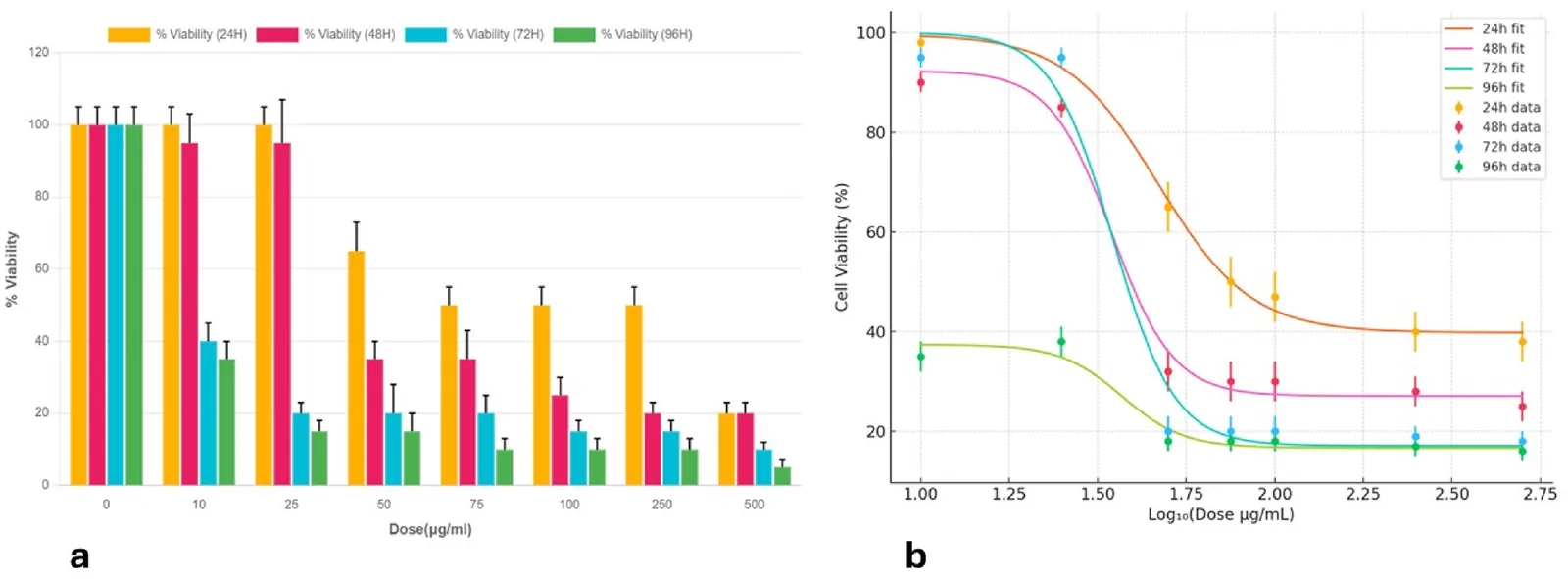
MTT assay showing HGF-1 cell viability after 24, 48, 72, and 96 h of incubation with different concentrations of *B. serrata* extract. (a) Bar chart showing cell viability according to extract concentration and incubation time. (b) IC50 dose-response curves. Data are presented as mean ± SD from three independent experiments (n = 3), with each experimental condition assessed in technical triplicate. MTT data were analyzed using two-way ANOVA with extract concentration and incubation time as factors, including the concentration × time interaction, followed by Tukey's multiple-comparisons test.

The IC50 of *B. serrata* extract is presented in Fig. 1. Concentrations up to 10 μg/mL maintained cell viability above 80% for up to 72 h. Therefore, concentrations of 2.5, 5, and 10 μg/mL were selected for further analysis. Two-way ANOVA demonstrated significant effects of extract concentration and incubation time on HGF-1 cell viability, as well as a significant concentration × time interaction (all p < 0.0001). Cell viability generally decreased with increasing extract concentration and prolonged incubation, although the magnitude of the time-dependent reduction varied according to extract concentration. A marked decline in cell viability was observed at higher extract concentrations, particularly with prolonged incubation.

### 8.2 Scratch assay

**Fig. 2:**
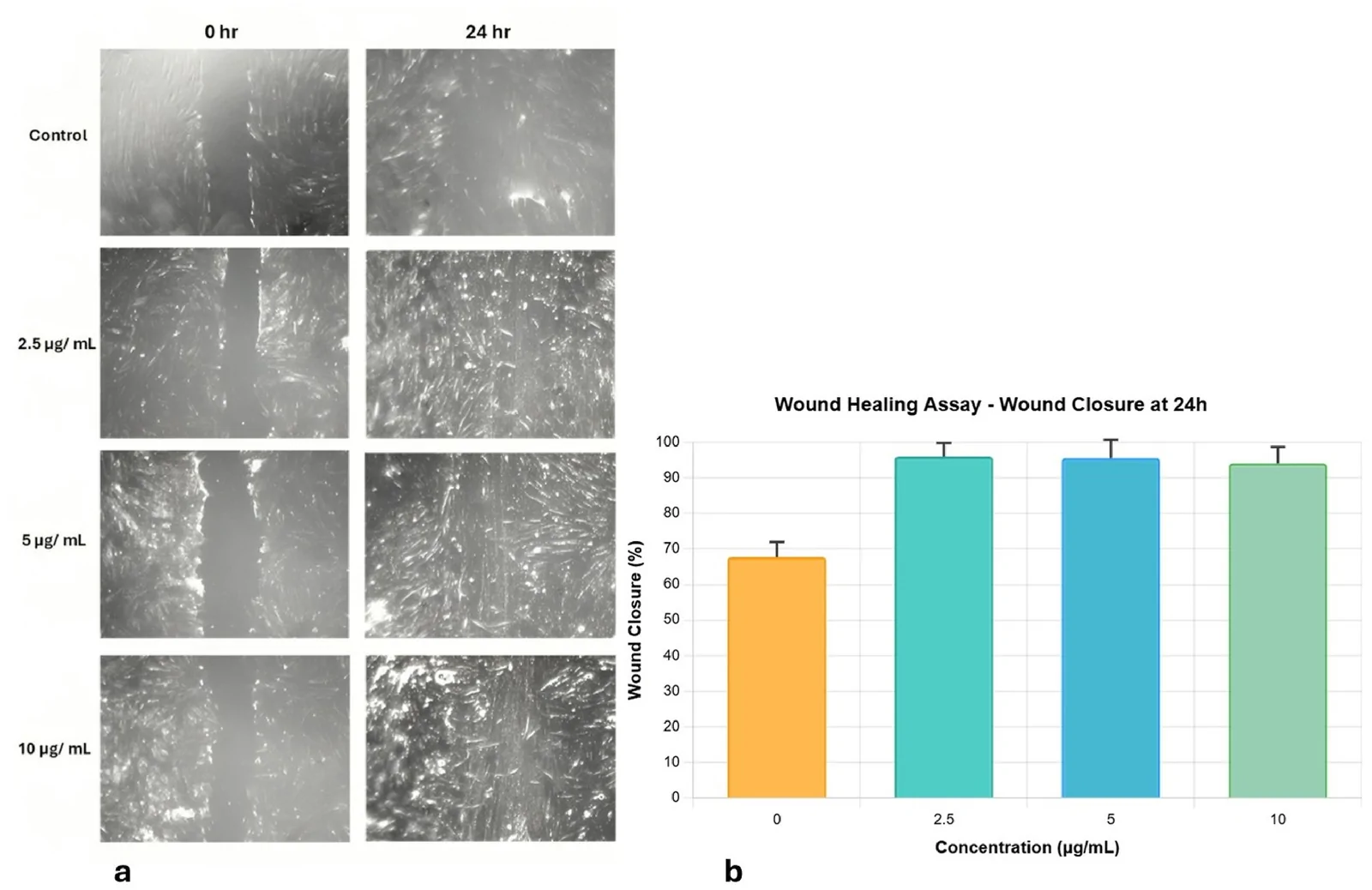
**Effect of *B. serrata* extract on HGF-1 scratch wound closure.** (a) Representative microscopic images of scratch wounds at 0 and 24 h after treatment. (b) Quantification of wound closure after 24 h of treatment with 0, 2.5, 5, and 10 μg/mL B*. serrata* extract. Data are presented as mean ± SD from three independent experiments (n = 3), each performed with three technical replicate wells per condition. The overall difference among groups was statistically significant (one-way ANOVA, p = 0.012), followed by Tukey's post hoc test. After 24 h, wound closure was 67.8% in the control group and 99.9%, 95.6%, and 94.1% in the 2.5, 5, and 10 μg/mL treatment groups, respectively.

Concentrations of 2.5, 5, and 10 μg/mL which maintained cell viability above 80%, were considered biocompatible and chosen for scratch assays. A significant overall difference in wound closure was observed among the experimental groups (one-way ANOVA, p = 0.012) (Fig. 2).

### 8.3 qRT-PCR analysis of gene expression

**Fig. 3:**
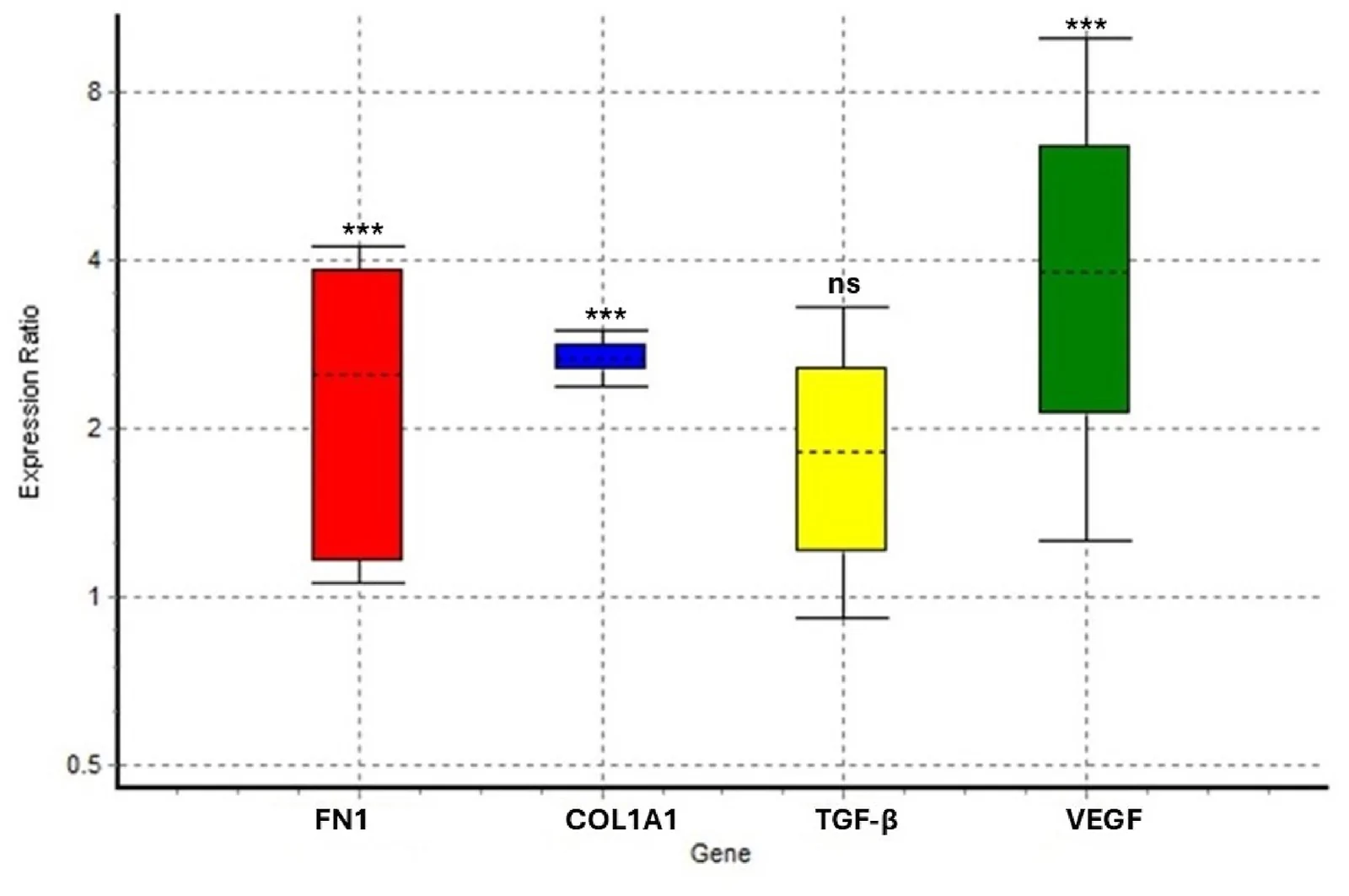
qRT-PCR analysis of wound healing-related gene expression in HGF-1 cells treated with *B. serrata* extract. Box plots show the relative expression levels of FN1, COL1A1, TGF-β, and VEGF compared with the untreated control. Relative expression levels were normalized to B2M and calculated using the ΔΔCt method. The dotted horizontal line indicates the control expression ratio of 1. Boxes represent the interquartile range, dashed lines indicate medians, and whiskers denote minimum and maximum values. ***P(H1) < 0.001; ns, not significant.

*B. serrata* extract significantly modulated wound healing gene expression in HGF-1 cells (Fig. 3, Table 1). COL1A1, FN1, and VEGF were significantly upregulated with fold changes of 2.68, 2.12, and 3.54, respectively (P < 0.001). TGF-β showed a non-significant increase (fold change = 1.74, P = 0.491). The B2M housekeeping gene served as the reference, maintaining stable expression across samples.

## 9 Discussion

The present study provides the first comprehensive evaluation of *Boswellia serrata* extract's effects on cellular and molecular behaviors of human gingival fibroblasts. Collectively, these findings indicate that *B*. *serrata* extract favorably influences cellular responses relevant to wound healing in human gingival fibroblasts, including cell viability, scratch wound closure, and wound healing-related gene expression.

While *B. serrata*'s anti-inflammatory and wound healing properties are well established on various cell types and disease models; oral research remains limited. The few existing studies have primarily focused on antimicrobial effects against oral pathogens. Attallah et al. demonstrated that *Boswellia sacra* extract reduced *Porphyromonas gingivalis* growth and inhibited biofilm formation.^12^ Similarly, Bakhtiari et al. showed that *B. serrata* hydro-alcoholic extracts effectively inhibited *Candida albicans* and *Streptococcus mutans*.^19^ Additionally, Sabra reported that *B. sacra* chewing gum reduced salivary bacterial counts, suggesting its potential as a natural oral hygiene intervention.^13^ While these studies demonstrate the antimicrobial efficacy of *Boswellia* species against various oral pathogens, their direct cellular effects on oral fibroblasts have not been explored.

The MTT assay results revealed that *B. serrata* extract concentrations up to 10 μg/mL maintained cell viability above 80% for up to 72 h, establishing an initial non-cytotoxic concentration range for further preclinical investigation. These biocompatibility findings align well with studies on other cell types, including colon fibroblasts^20^ and endothelial cells^10^ demonstrating similar safety profiles at comparable concentrations. This consistency across different cell populations suggests that the present HGF findings are in line with biocompatibility responses reported in other cell models. Notably, the effective concentrations in our study were significantly lower than those required for antimicrobial activity reported by Bakhtiari et al. (50-100 mg/mL),^19^ suggesting that cellular effects on human gingival fibroblasts occur at much lower, potentially safer concentrations than those needed for direct antimicrobial action.

Our work is the first to establish specific non-cytotoxic concentrations for human gingival fibroblasts. The observed dose-dependent cytotoxicity at higher concentrations (≥25 μg/mL) aligns with established principles that natural compounds can exhibit both therapeutic and toxic effects depending on dosage, emphasizing the importance of concentration optimization in therapeutic applications. The scratch assay demonstrated enhanced wound closure at all tested concentrations. Although the highest numerical response was observed at 2.5 μg/mL(99.9% closure), no formal statistical comparison established superiority over 5 or 10 μg/mL; therefore, this finding should be interpreted descriptively rather than as evidence of an optimal concentration. The numerically greater response observed at the lower concentration may indicate a concentration-dependent pattern that warrants further investigation in dedicated dose-response studies.^21^ Given that HGFs possess multi-differentiation and self-renewal capacities^18^ and HGFs are among the first cells to repopulate gingival wound sites and play central roles in tissue repair, *B. serrata* extract could effectively enhance the natural regenerative capacity of periodontal tissues at minimal, non-cytotoxic doses.

Regarding the wound healing effects of *Boswellia serrata*, our study demonstrates its effect on HGF-1 scratch wound closure and the upregulation of key genes involved in wound healing. qRT-PCR analysis revealed increased expression of critical ECM-related genes, including COL1A1 (2.68-fold), FN1 (2.12-fold), and VEGF (3.54-fold), following treatment with *B. serrata* extract. These molecular findings were observed alongside enhanced scratch wound closure. Importantly, these effects occurred at concentrations (2.5, 5, and 10 μg/mL) that maintained cell viability above 80%, suggesting that the observed wound healing-related responses occurred within a non-cytotoxic concentration range. Although the effective concentrations identified in this study, particularly 2.5–10 μg/mL, provide an initial biocompatible range for human gingival fibroblasts, direct translation to clinical dosing should be interpreted cautiously. In vivo effectiveness would depend on the local delivery method, extract retention at the wound site, release kinetics, tissue penetration, salivary clearance, and formulation stability. Therefore, these concentrations should be considered a starting point for future topical formulation and preclinical dose-response studies rather than definitive clinical doses. Scratch wound closure can result from both fibroblast migration and proliferation. Because a proliferation inhibitor was not used in the present study, the relative contribution of each process to the observed wound closure could not be determined.

The marked upregulation of VEGF (3.54-fold) represents the highest fold-change among the tested genes. Given the established role of VEGF in angiogenesis and vascular remodeling, this finding suggests that angiogenesis-related signaling may warrant further investigation. However, angiogenesis itself was not directly assessed in the present study. Enhanced angiogenesis is particularly critical in periodontal regeneration, where sufficient vascularization determines the success of tissue repair and the long-term stability of regenerated tissues.^22^ Notably, our findings are consistent with *B. serrata*'s established role in treating inflammatory diseases, where angiogenesis and tissue repair are crucial to resolution.^23^ The significant upregulation of FN1 (2.12-fold) is consistent with its established role in cellular adhesion, migration, and provisional matrix formation during early wound healing. This upregulation was observed alongside enhanced scratch wound closure. Similarly, the increased expression of COL1A1 (2.68-fold) is consistent with an increased ECM-related gene-expression response, given the established structural role of type I collagen in healing tissues. Interestingly, however, recent studies on intestinal fibroblasts have shown that *B. serrata* reduced fibrotic markers, including FN1 and COL1A1, under inflammatory conditions.^20^ This contrast implies that *B. serrata* may exert context-dependent effects—promoting regeneration in healthy tissue while mitigating fibrosis in pathological states.

Interestingly, TGF-β expression showed only modest upregulation (1.74-fold) without statistical significance. The non-significant increase in TGF-β indicates that the observed cellular and gene-expression responses cannot be attributed to a significant change in TGF-β expression under the conditions evaluated in this study. Given the established role of TGF-β in fibroblast activation, ECM synthesis, myofibroblast differentiation, and scar formation,^24^ the absence of significant upregulation may indicate that the observed wound healing-related responses were not primarily mediated by marked TGF-β activation and may involve alternative pathways. This is supported by findings that *B. serrata* promotes oral healing through inflammation resolution via PPARγ (peroxisome proliferator-activated receptor gamma)-dependent pathways^25^ and non-classical targets such as mPGES-1 (microsomal prostaglandin E synthase-1) and cathepsin G, rather than solely through leukotriene or TGF-β suppression.^26^^,^^27^

Taken together, these findings suggest that the wound healing-related cellular and molecular responses observed following *B. serrata* treatment on gingival fibroblasts may involve multiple complementary signaling mechanisms. Boswellic acids have been reported to inhibit 5-lipoxygenase and leukotriene-mediated inflammatory responses, which may help reduce inflammatory conditions that impair periodontal repair.^9^, ^10^, ^11^^,^^15^^,^^16^ In addition, modulation of NF-κB and MAPK-related pathways may influence fibroblast migration, survival, and extracellular matrix remodeling.^15^^,^^16^ The previously reported involvement of PPARγ-mediated inflammation resolution may also contribute to a tissue environment favorable for wound healing.^25^ In the present study, the marked upregulation of VEGF further suggests a possible contribution of angiogenesis-related signaling to the regenerative response.^22^ However, these pathways were not directly examined in this study and should be validated in future mechanistic investigations.

Our findings provide scientific validation for traditional applications of *B. serrata* in oral health, including strengthening gums, treating inflammatory conditions, and promoting wound healing. Beyond its well-documented anti-inflammatory, antioxidant, and antimicrobial activities, our results suggest additional molecular mechanisms through which *B. serrata* may enhance gingival fibroblast function and wound repair.

The demonstrated upregulation of multiple wound healing-related genes suggests that *B. serrata* could have potential as an adjunctive approach in periodontal treatment protocols. The observed enhancement of scratch wound closure and increased VEGF expression supports potential relevance for various periodontal interventions, including surgical procedures, guided tissue regeneration, and management of periodontal defects.

## 10 Limitations and future directions

This study has several limitations. First, the experiments were performed in vitro using HGF-1 cells; therefore, the findings may not fully reproduce the complexity of periodontal wound healing in vivo, where epithelial cells, immune cells, periodontal ligament cells, osteoblasts, vascular components, saliva, and microbial biofilms interact dynamically. Second, only one gingival fibroblast cell line was used, which may not capture donor-to-donor biological variability. Third, although the plant material was obtained from a certified herbal medicine supplier with established quality-control procedures, a voucher specimen number was not available. In addition, the crude methanolic extract was not chemically standardized or quantitatively characterized for boswellic acids or other constituents. Variation in phytochemical composition according to source and extraction conditions may therefore limit compositional reproducibility and attribution of the observed effects to specific active constituents. Fourth, this study assessed gene expression changes, but protein-level validation of COL1A1, FN1, VEGF, and TGF-β was not performed. Fifth, the MTT assay reflects cellular metabolic activity and was used as an indicator of cell viability; therefore, it does not directly establish changes in cell proliferation. In addition, scratch wound closure may result from both fibroblast migration and proliferation. Because a proliferation inhibitor was not used, the relative contributions of these processes to the observed wound closure could not be distinguished. Finally, pharmacokinetic behavior, local tissue retention, release kinetics, and safety in animal or clinical models remain unknown. Future studies should evaluate purified boswellic acid fractions, protein expression, pathway activation, periodontal co-culture or biofilm models, and animal or clinical validation before clinical application.

## 11 Conclusion

Within the limitations of this in vitro study, *Boswellia serrata* extract at concentrations ≤10 μg/mL maintained HGF-1 cell viability and enhanced scratch wound closure. Treatment was also associated with increased expression of COL1A1, FN1, and VEGF, whereas the change in TGF-β expression was not statistically significant. These findings support further preclinical investigation of *B. serrata* in periodontal wound-healing models.

## Use of human subjects and animals

This study utilized human gingival fibroblast cell line (HGF-1) obtained from a national cell bank.

No human participants were directly involved in this research.

No animals were used in this study.

## Patient's/guardian's consent

Not applicable.

## Ethical clearance

Ethics approval has been obtained from the Ethics Committee of Islamic Azad University, Borujerd Branch, with approval code IR.IAU.B.REC.1402.078.

## Ethics approval

This study was approved by the Ethics Committee of Islamic Azad University, Borujerd Branch.

Approval code: IR.IAU.B.REC.1402.078.

## Funding

This research was self-funded by the authors.

## Declaration of competing interest

The authors declare that they have no known competing financial interests or personal relationships that could have appeared to influence the work reported in this paper.
